# Resonance assignment of the intrinsically disordered actin-binding region of Drebrin

**DOI:** 10.1007/s12104-025-10239-0

**Published:** 2025-06-14

**Authors:** Soma Varga, Julie Maibøll Kaasen, Zoltán Gáspári, Bálint Ferenc Péterfia, Frans A. A. Mulder

**Affiliations:** 1 Faculty of Information Technology and Bionics, Pázmány Péter Catholic University, Práter Street 50/A, Budapest, 1083 Hungary; 2 Institute of Biochemistry, Johannes Kepler University, Altenberger Straße 69, Linz, 4040 Austria

**Keywords:** Intrinsically disordered protein, Carbonyl-carbonyl J-coupling, Drebrin, Actin-binding

## Abstract

Drebrin (developmentally regulated brain protein) is a vital component of the Postsynaptic Density (PSD). It performs important biological roles as it interacts with the postsynaptic protein Homer and anchors the complete protein network to the cellular skeleton through actin filaments. Drebrin contains unique structural elements including an evolutionarily unconventional actin-depolymerizing factor homology (ADFH) domain that has lost its strong actin-binding ability, and a Single Alpha-Helix (SAH) motif harbored by long flexible regions. In vivo studies have suggested that a disordered segment in Drebrin plays a key role in binding filamentous actin, yet the atomic-level characterization of the binding interface between these proteins has not been reported. To bridge this gap, we designed the intrinsically disordered construct D233 and employed 3D (HN)CO(CO)NH NMR spectroscopy to accomplish a near-complete backbone resonance assignment. This work serves as an essential step toward a detailed structural and functional investigation of the interaction between Drebrin and F-Actin.

## Biological context

**Fig. 1:**
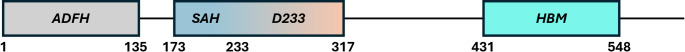
Schematic depiction of Drebrin and its regions. ADFH: Actin-Depolymerizing Factor Homology domain, SAH: Single Alpha-Helix, HBM: Homer-binding Motif. Exact boundaries between the SAH and neighboring motifs are not yet explored and there might be a transition between them

Drebrin (Uniprot ID Q16643) is an actin cytoskeleton-organizing protein (Pérez-Martínez et al. 2010) that plays a crucial part in the morphogenesis and organization of dendritic spines (Lin et al. 2023). Previous studies have described the actin-bundling (Li et al. 2019), the actin-binding (Hayashi et al. 1999) and the actin-depolymerizing (Mikati et al. 2013) properties of Drebrin, showcasing its complex role in Postsynaptic Density (PSD). Furthermore, it has been demonstrated that Drebrin only interacts with filamentous (F) actin and not with the monomeric (G) form (Lappalainen et al. 1998). To date, there is no consensus on the exact domain organization and the biological role of many unique structural motifs in Drebrin (Fig. 1). Residues 173–238 have been proposed to form a Single Alpha-Helix (Kovács et al. 2018) opposing the earlier suggestion of a putative coiled-coil motif (Worth et al. 2013). The Actin-Binding Domain (ABD) has been investigated in several in vivo studies (Hayashi and Shirao 1999; Biou et al. 2008; Ivanov et al. 2009), as well as in vitro, by low-resolution analytical methods such as cosedimentation assays. Results from these studies do not concur and suggest the location of Drebrin ABD in different sequential positions, including the primary binding site between residues 233–300 or 233–317, with *K*_D_ values between 6.2 and 8.2 µM (Grintsevich et al. 2010). Worth et al. further suggested the possibility of cooperative binding involving N-terminal parts of Drebrin as well. Secondary structure analysis from circular dichroism data by Grintsevich et al. suggests the ABD to be highly disordered.

In this study we designed a Drebrin construct containing residues 233–317, coined D233, suitable for bacterial expression, purification, and NMR spectroscopy.

Detailed investigation of the Actin binding mechanism of Drebrin bears complex challenges on behalf of both partners. F-Actin is a dynamic double-helical complex with heterogeneous size distribution, unsuitable for X-ray crystallography (Grintsevich 2017). The predicted flexible nature of our D233 construct and the high preponderance of charged residues prompted us to use NMR spectroscopy, which is a powerful method to study disordered proteins (Schiavina et al. 2024). The narrow amide proton chemical shift dispersion (Mäntylahti et al. 2009; Motáčková et al. 2010; Nováček et al. 2011) presents one of the main challenges for disordered proteins. Several 4–5 or even 6 dimensional NMR methods have been developed in order to overcome this limitation, although they result in long experiment time and complex data interpretation (Murrali et al. 2018; Tossavainen et al. 2020). In this work we demonstrate the successful application of 3D (HN)CO(CO)NH NMR spectroscopy (Yoshimura et al. 2015; Kulminskaya et al. 2016) which tremendously reduced the number of multidimensional NMR experiments required and vastly accelerated the assignment procedure.

## Methods and experiments

### Cloning, expression, and purification

The DNA sequence of the full Drebrin protein (from *Homo sapiens* Uniprot Q16643) was a gift from Phillip Gordon-Weeks (Addgene plasmid #40362; http://n2t.net/addgene:40362;RRID: Addgene_40362) and used as template for cloning residues 233–317 into NdeI and HindIII sites of a modified pET-15b vector (Novagen) with N-terminal 6xHis-tag and TEV cleavage site (ENLYFQG) using primers 5’-aa aaa cat atg GAG GAG CAC AGG AGG AAA C-3’ (D233 forward) 3’- aaa AAG CTT CTA TCG ATG GTT GAA GGG CG-5’ (D233 reverse). We used BL21 (DE3) *E. coli* cells for expressing the protein in M9 minimal medium enriched with 1.5 g/l ^15^NH_4_Cl and 2.5 g/l ^13^C D-glucose as the only nitrogen and carbon sources. After reaching OD_600_ = 1.0, expression was induced with 1 mM isopropyl β-D-1-thiogalactopyranoside (IPTG), and cells were incubated for 3 h at 37 °C before harvesting by centrifugation. The cells were extracted by sonication using lysis buffer (300 mM NaCl, 43 mM Na_2_HPO_4_, 7 mM NaH_2_PO_4_, 5 mM β-Mercaptoethanol, 1 mM AEBSF protease inhibitor cocktail, pH 7.4), and the lysate was centrifuged at 2423xg for 20 min at 4 °C. The resulting supernatant was filtered through a 0.45 μm membrane prior to chromatography. A Ni^2+^-affinity column (Bio-Scale 5 ml Nuvia IMAC) equilibrated with binding buffer (300 mM NaCl, 43 mM Na_2_HPO_4_, 7 mM NaH_2_PO_4_, 5 mM β-Mercaptoethanol, pH 7.4) was used to purify His-tagged D233. D233 was eluted with an imidazole gradient / 500 mM imidazole, followed by the cleavage of the His-tag with Tobacco Etch Virus (TEV) protease. Prior to ion exchange chromatography, the buffer was exchanged to a low salt binding buffer (20 mM NaCl, 35 mM NaH_2_PO_4_, 15 mM Na_2_HPO_4_, 5 mM β-Mercaptoethanol, pH 6.5), followed by purification using a Q column (Bio-Scale Mini Macro-Prep). The protein was collected in the flow through fraction and concentrated using Amicon Ultra Centrifugal Filter (3 kDa MWCO). The D233 protein construct used for NMR experiments had the following sequence: gshmEEHRRK QQTLEAEEAK RRLKEQSIFG DHRDEEEETH MKKSESEVEE AAAIIAQRPD NPREFFKQQE RVASASAGSC DVPSPFNHR. Assuming natural isotopic abundance, the molecular weight of D233 is 10.32 kDa, which matched migration on SDS-PAGE.

### Backbone assignment experiments

**Fig. 2:**
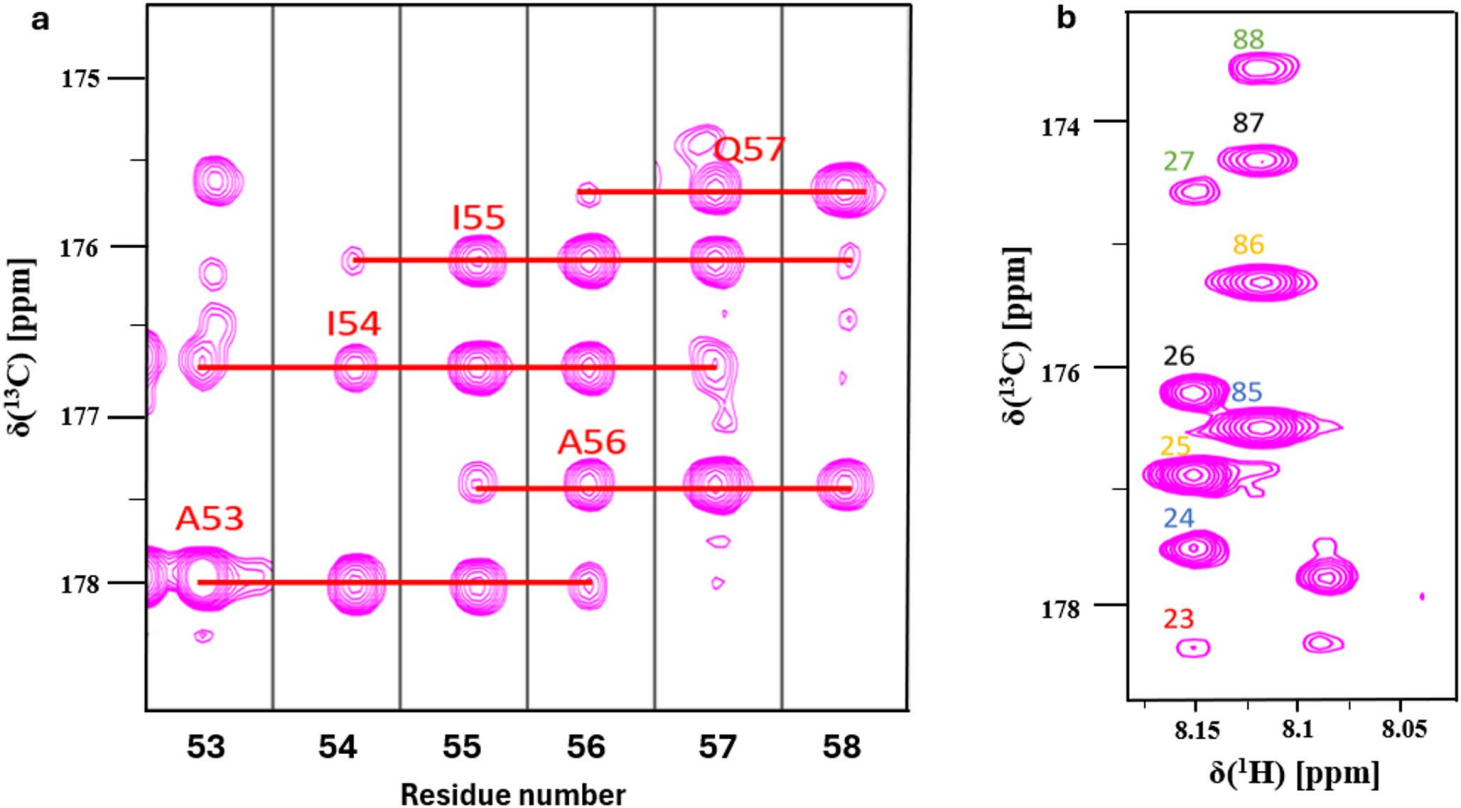
(**a**) Strip plots for adjacent residues (plotted at the amide ^1^H regions for A53 to R58) of uniformly ^13^C,^15^N-labelled D233 from 3D (HN)CO(CO)NH experiment. (**b**) A zoomed F1-F3 region of the 3D (HN)CO(CO)NH spectrum showing the correlation between multiple carbonyl signals (green: i + 1, black: i, orange: i-1, blue: i-2, red: i-3). In highly resolved areas it is possible to recognize up to 5 carbonyl correlations following each other in the sequence

The ^15^N, ^13^C labelled D233 sample in NMR buffer (50 mM NaCl, 17 mM NaH_2_PO_4_, 3 mM Na_2_HPO_4_, pH 6.0, 5 mM TCEP, 10% (v/v) D_2_O) was transferred to 5 mm NMR tubes with a final protein concentration of 0.3 mM. Data were collected at 25 °C, using a 700 MHz Bruker Avance III NMR spectrometer equipped with a cryogenically cooled TCI probe head with single-axis field gradient. DSS (2,2-dimethyl-2-silapentane-5-sulfonate sodium salt) was used for direct ^1^H chemical shift referencing, while ^15^N and ^13^C chemical shifts were referenced indirectly following the IUPAC definition (Markley et al. 1998). The backbone assignment strategy made use of long-range carbonyl-carbonyl correlations to bypass the chemical shift degeneracy of amide protons; The 3D (HN)CO(CO)NH experiment takes advantage of the favorable relaxational properties of ^13^C carbonyl nuclei, due to their rapid segmental dynamics in disordered segments. This enables a particularly long acquisition using a semi-constant time t_1_-evolution, in the ^13^C indirect dimension, thereby increasing the resolution. The MOCCA-XY16 pulse scheme allows an extremely long isotropic mixing time making it possible to correlate several adjacent residues. In our experiments, an isotropic mixing time of 500 ms was used, therefore we were able to obtain correlations between up to 5 neighboring amino acids, as illustrated in Fig. 2a. The experiment also provided some directional information encoded in the peak intensities (Fig. 2b), with the less intense signals being further removed in sequence from the NH group in question. The assignment procedure was further aided by the information on amino acid type obtained from a 3D (H)CC(CO)NH experiment. To further support the assignment, HNCO and HN(CA)CO experiments were also recorded.

### Assignments and data deposition

**Fig. 3:**
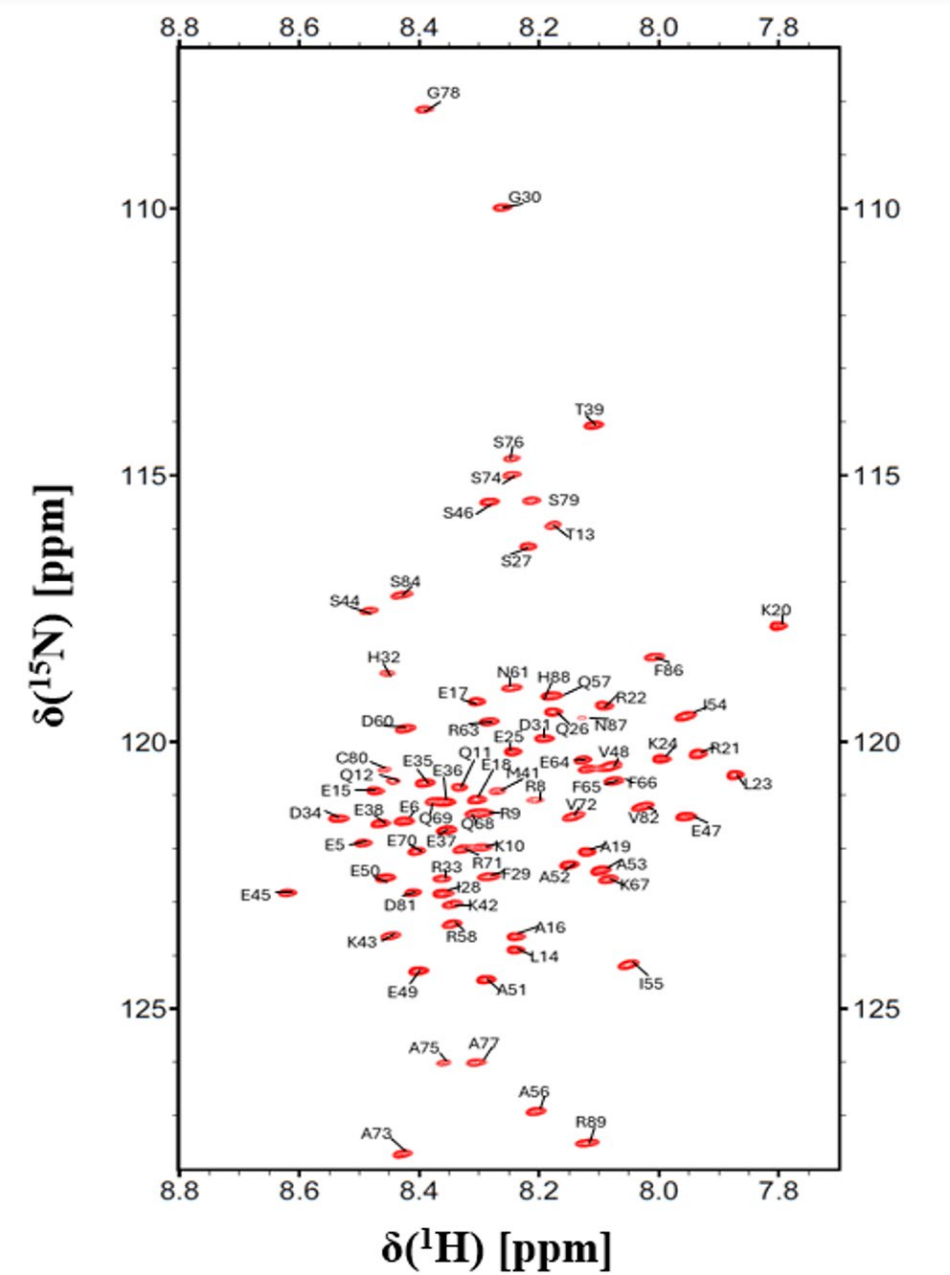
^1^H-^15^N HSQC spectrum of the D233 construct in 50 mM NaCl, 17 mM NaH_2_PO_4_, 3 mM Na_2_HPO_4_, pH 6.0, 5 mM TCEP measured at 25 °C

**Fig. 4:**
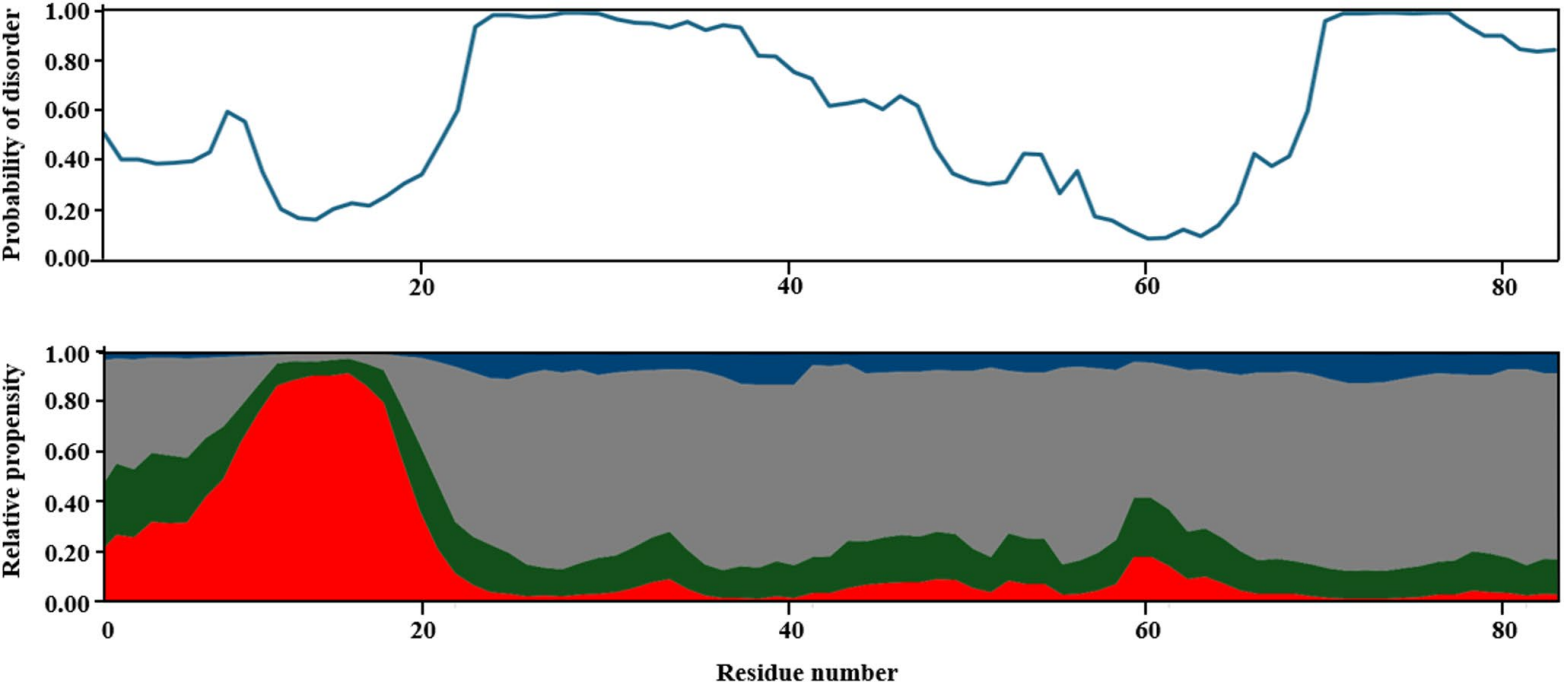
Comparison of disorder prediction according to ODiNpred (top panel, blue line) with secondary structure propensities obtained via CheSPI (bottom panel; red area: α-helix, green area: turn, grey area: disordered, blue area: extended)

The results of the backbone assignment can be considered complete (Fig. 3). From the 85 amino acids, 81 have been assigned unambiguously. The amide backbone chemical shifts of the four proline residues are missing due to the lack of H^N^ protons. Aliphatic ^13^C side chain resonances have been also included in the deposition. Cβ chemical shifts of prolines all appear close to 32 ppm suggesting the presence of *trans* isomers. 96% of CO, 93% of Cα and 92% of Cβ resonances were assigned. The aliphatic carbon chemical shifts were used to derive secondary structure propensities with CheSPI (Nielsen and Mulder 2021), and compared with the predicted propensities by ODiNpred (Dass et al. 2020), based on amino acid sequence alone (Fig. 4). The assignments obtained for D233 have been deposited in the BMRB with the access number 52895.

The relative propensities revealed by CheSPI align well with the ODiNpred disorder prediction. The only notable difference between the results can be observed spanning residues 50 and 70, where ODINpred suggests a slightly longer ordered region. In comparison, CheSPI results indicate only a shorter segment with minor secondary structure propensity, harbored by disordered regions, which appears to be more accurate when looking at the chemical shift values. Following the first 20 residues, the construct adapts a mostly disordered conformation with a minor region of decreased mobility around residue 60. The observed high helical content at the N-terminus might be considered as the C-terminal extension of the Single-Alpha Helix, as in the full protein, the presence of a longer stable helix is plausible. This raises further questions about the exact domain organization of Drebrin, highlighting the importance of investigating the actin-binding region of Drebrin at an atomic level.

## Data Availability

The assignments obtained for D233 have been deposited in the BMRB with the access number 52895.
